# Efficient photocatalytic carbon monoxide production from ammonia and carbon dioxide by the aid of artificial photosynthesis†
†Electronic supplementary information (ESI) available: Experimental details, calculations and characterizations. See DOI: 10.1039/c7sc01851g



**DOI:** 10.1039/c7sc01851g

**Published:** 2017-06-19

**Authors:** Zeai Huang, Kentaro Teramura, Hiroyuki Asakura, Saburo Hosokawa, Tsunehiro Tanaka

**Affiliations:** a Department of Molecular Engineering , Graduate School of Engineering , Kyoto University , Kyoto 615-8510 , Japan . Email: teramura@moleng.kyoto-u.ac.jp ; Email: tanakat@moleng.kyoto-u.ac.jp; b Elements Strategy Initiative for Catalysts and Batteries , Kyoto University , Kyoto 615-8510 , Japan

## Abstract

Ammonium bicarbonate (NH_4_HCO_3_) was generated by the absorption of carbon dioxide (CO_2_) into an aqueous solution of ammonia (NH_3_). NH_4_HCO_3_ was successfully used to achieve highly efficient photocatalytic conversion of CO_2_ to carbon monoxide (CO). NH_3_ and/or ammonium ions (NH_4_^+^) derived from NH_4_HCO_3_ in aqueous solution were decomposed into nitrogen (N_2_) and hydrogen (H_2_). Stoichiometric amounts of the N_2_ oxidation product and the CO and H_2_ reduction products were generated when the photocatalytic reaction was carried out in aqueous NH_4_HCO_3_ solution. NH_3_ and/or NH_4_^+^ functioned as electron donors in the photocatalytic conversion of CO_2_ to CO. A CO formation rate of 0.5 mmol h^–1^ was obtained using 500 mg of catalyst (approximately 7500 ppm) in ambient conditions (303 K, 101.3 kPa). Our results demonstrated that NH_4_HCO_3_ is a novel inorganic sacrificial reagent, which can be used to increase the efficiency of photocatalytic CO production to achieve one step CO_2_ capture, storage and conversion.

## Abstract 2

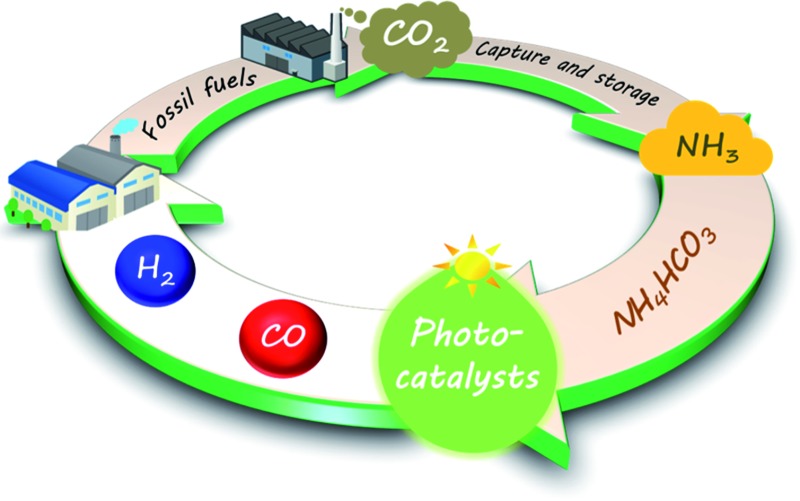
NH_4_HCO_3_ was determined to be an effective electron donor for the photocatalytic conversion of CO_2_, whereby CO_2_ can be captured, stored, and efficiently converted into CO.

## Introduction

The production of chemical feedstocks and hydrocarbon fuels from CO_2_ is a promising approach to alleviate the global energy crisis and global warming.^1^ Conversion of CO_2_ to CO using clean and renewable solar energy is the first step to store energy in chemicals because CO can be further converted into other highly valuable chemicals using the Fischer–Tropsch process.^2^ A variety of heterogeneous and homogeneous photocatalysts have been reported to achieve the conversion of CO_2_ to CO.^3–5^ However, the formation rate of CO has been limited to a few tens of μmol h^–1^ or hundreds of μmol h^–1^ g^–1^ because of the high energy barrier to CO_2_ reduction and inefficient light utilization.^6,7^ Furthermore, CO_2_ is not easily adsorbed onto catalytic surfaces nor activated by photoirradiation because of its high thermodynamic stability. This further reduces the efficiency of the photocatalytic conversion of CO_2_.

Water (H_2_O) is widely used as an electron donor in the photocatalytic conversion of CO_2_ to CO.^7–12^ However, the overall water splitting into H_2_ and O_2_ is more thermodynamically favorable than the reduction of CO_2_ in aqueous solution. Hence, CO_2_ reduction competes with overall water splitting. Moreover, the solubility of CO_2_ in pure H_2_O is only 0.033 mol L^–1^ (at 298 K and 101.3 kPa),^13^ which further limits the efficiency of CO_2_ conversion by H_2_O using heterogeneous photocatalysts. Therefore, it would be meaningful to find a readily available, highly efficient, and abundant in nature and industries electron donor (sacrificial reagent) other than water for the photocatalytic conversion of CO_2_. NH_3_ and NH_4_^+^ in aqueous solution can be readily oxidized to N_2_, NO_2_^–^, and NO_3_^–^ using a photocatalyst.^14–17^ The decomposition of aqueous NH_3_ to H_2_ and N_2_ requires a standard Gibbs free energy change Δ*G*° of 18 kJ mol^–1^ (eqn (1)).^18^ This is significantly smaller than that required for the decomposition of H_2_O to H_2_ and O_2_ (237 kJ mol^–1^; eqn (2)).

1 NH_3_(aq) → 

<svg xmlns="http://www.w3.org/2000/svg" version="1.0" width="16.000000pt" height="16.000000pt" viewBox="0 0 16.000000 16.000000" preserveAspectRatio="xMidYMid meet"><metadata>
Created by potrace 1.16, written by Peter Selinger 2001-2019
</metadata><g transform="translate(1.000000,15.000000) scale(0.005147,-0.005147)" fill="currentColor" stroke="none"><path d="M400 2680 l0 -40 -40 0 -40 0 0 -40 0 -40 -80 0 -80 0 0 -80 0 -80 80 0 80 0 0 80 0 80 80 0 80 0 0 40 0 40 280 0 280 0 0 -80 0 -80 40 0 40 0 0 -80 0 -80 -40 0 -40 0 0 -40 0 -40 -120 0 -120 0 0 -40 0 -40 -80 0 -80 0 0 -40 0 -40 80 0 80 0 0 -40 0 -40 120 0 120 0 0 -40 0 -40 40 0 40 0 0 -80 0 -80 -80 0 -80 0 0 -80 0 -80 -240 0 -240 0 0 40 0 40 -80 0 -80 0 0 80 0 80 -80 0 -80 0 0 -80 0 -80 80 0 80 0 0 -40 0 -40 40 0 40 0 0 -40 0 -40 320 0 320 0 0 40 0 40 40 0 40 0 0 80 0 80 80 0 80 0 0 80 0 80 -40 0 -40 0 0 40 0 40 -40 0 -40 0 0 40 0 40 -120 0 -120 0 0 40 0 40 120 0 120 0 0 40 0 40 40 0 40 0 0 40 0 40 40 0 40 0 0 80 0 80 -40 0 -40 0 0 80 0 80 -40 0 -40 0 0 40 0 40 -360 0 -360 0 0 -40z M2640 2680 l0 -40 -40 0 -40 0 0 -40 0 -40 -40 0 -40 0 0 -40 0 -40 -40 0 -40 0 0 -40 0 -40 -40 0 -40 0 0 -40 0 -40 -40 0 -40 0 0 -40 0 -40 -40 0 -40 0 0 -40 0 -40 -40 0 -40 0 0 -40 0 -40 -40 0 -40 0 0 -40 0 -40 -40 0 -40 0 0 -40 0 -40 -40 0 -40 0 0 -40 0 -40 -40 0 -40 0 0 -40 0 -40 -40 0 -40 0 0 -40 0 -40 -40 0 -40 0 0 -40 0 -40 -40 0 -40 0 0 -40 0 -40 -40 0 -40 0 0 -40 0 -40 -40 0 -40 0 0 -40 0 -40 -40 0 -40 0 0 -40 0 -40 -40 0 -40 0 0 -40 0 -40 -40 0 -40 0 0 -40 0 -40 -40 0 -40 0 0 -40 0 -40 -40 0 -40 0 0 -40 0 -40 -40 0 -40 0 0 -40 0 -40 -40 0 -40 0 0 -40 0 -40 -40 0 -40 0 0 -40 0 -40 -40 0 -40 0 0 -40 0 -40 -40 0 -40 0 0 -40 0 -40 -40 0 -40 0 0 -40 0 -40 -40 0 -40 0 0 -40 0 -40 -40 0 -40 0 0 -40 0 -40 -40 0 -40 0 0 -40 0 -40 -40 0 -40 0 0 -40 0 -40 -40 0 -40 0 0 -40 0 -40 -40 0 -40 0 0 -40 0 -40 80 0 80 0 0 40 0 40 40 0 40 0 0 40 0 40 40 0 40 0 0 40 0 40 40 0 40 0 0 40 0 40 40 0 40 0 0 40 0 40 40 0 40 0 0 40 0 40 40 0 40 0 0 40 0 40 40 0 40 0 0 40 0 40 40 0 40 0 0 40 0 40 40 0 40 0 0 40 0 40 40 0 40 0 0 40 0 40 40 0 40 0 0 40 0 40 40 0 40 0 0 40 0 40 40 0 40 0 0 40 0 40 40 0 40 0 0 40 0 40 40 0 40 0 0 40 0 40 40 0 40 0 0 40 0 40 40 0 40 0 0 40 0 40 40 0 40 0 0 40 0 40 40 0 40 0 0 40 0 40 40 0 40 0 0 40 0 40 40 0 40 0 0 40 0 40 40 0 40 0 0 40 0 40 40 0 40 0 0 40 0 40 40 0 40 0 0 40 0 40 40 0 40 0 0 40 0 40 40 0 40 0 0 40 0 40 40 0 40 0 0 40 0 40 40 0 40 0 0 40 0 40 40 0 40 0 0 40 0 40 40 0 40 0 0 40 0 40 40 0 40 0 0 40 0 40 40 0 40 0 0 80 0 80 -40 0 -40 0 0 -40z M2080 1240 l0 -40 -80 0 -80 0 0 -40 0 -40 -80 0 -80 0 0 -80 0 -80 80 0 80 0 0 80 0 80 80 0 80 0 0 40 0 40 160 0 160 0 0 -40 0 -40 80 0 80 0 0 -200 0 -200 -80 0 -80 0 0 -40 0 -40 -80 0 -80 0 0 -40 0 -40 -80 0 -80 0 0 -40 0 -40 -80 0 -80 0 0 -40 0 -40 -40 0 -40 0 0 -40 0 -40 -40 0 -40 0 0 -160 0 -160 480 0 480 0 0 40 0 40 -400 0 -400 0 0 120 0 120 40 0 40 0 0 40 0 40 40 0 40 0 0 40 0 40 80 0 80 0 0 40 0 40 80 0 80 0 0 40 0 40 80 0 80 0 0 40 0 40 80 0 80 0 0 200 0 200 -80 0 -80 0 0 40 0 40 -80 0 -80 0 0 40 0 40 -160 0 -160 0 0 -40z"/></g></svg>

H_2_(g) + ½N_2_(g) Δ*G*° = 18 kJ mol^–1^

2 H_2_O(l) → H_2_(g) + ½O_2_(g) Δ*G*° = 237 kJ mol^–1^

Because the photocatalytic oxidation of NH_3_ and NH_4_^+^ is significantly more favorable than the oxidation of H_2_O to O_2_,^18^ it is possible to use NH_3_ and NH_4_^+^ as electron donors in the photocatalytic conversion of CO_2_. Moreover, NH_3_ has been considered for use as an efficient post-combustion CO_2_ capture and storage (CCS) reagent because of its high absorption efficiency and loading capacity.^19^ The absorption and capture of CO_2_ by an aqueous solution of NH_3_ results in the formation of NH_4_HCO_3_.^20^ Other basic species, such as NaHCO_3_ and KHCO_3_, have been used to increase the solubility of CO_2_ in aqueous solutions.^21,22^ Previous reports have suggested that dissolved CO_2_, rather than bicarbonate (HCO_3_^–^) or carbonate (CO_3_^2–^) ions, is the active species in the reduction of CO_2_.^23,24^ Correspondingly, the conversion of CO_2_ and/or the selectivity toward CO evolution have been significantly enhanced by the presence of bases in both photocatalytic (PC) and photoelectrochemical (PEC) cell systems.^25,26^ In the present study, we designed the use of NH_4_HCO_3_ for the efficient photocatalytic conversion of CO_2_ to CO in H_2_O.

## Results and discussion

Flux-mediated crystal growth method shows the advantage of the synthetic control over particle sizes, morphologies, and surface features comparing with that of solid-state reaction method (SSR).^27^ Modification of these features as a function of flux conditions have been reported to show significant enhancements in both water splitting and CO_2_ photoreduction.^11,27,28^ Sr_2_KTa_5_O_15_ has been reported to show good activity and selectivity toward CO evolution when used as a photocatalyst in the conversion of CO_2_ by H_2_O in our previous work.^29^ In the present study, Sr_2_KTa_5_O_15_ was fabricated by a modified flux method, using a mixture of NaCl and KCl as the flux. The resultant catalyst was confirmed to have tetragonal tungsten bronze (TTB) structure (Fig. S1A†), and its real chemical formula was determined to be Sr_1.6_K_0.35_Na_1.45_Ta_5_O_15_ using ICP-OES. Its morphology was observed by SEM, and was found to consist of a mixture of nanorods and nanoparticles (Fig. S1B†).

**Fig. 1:**
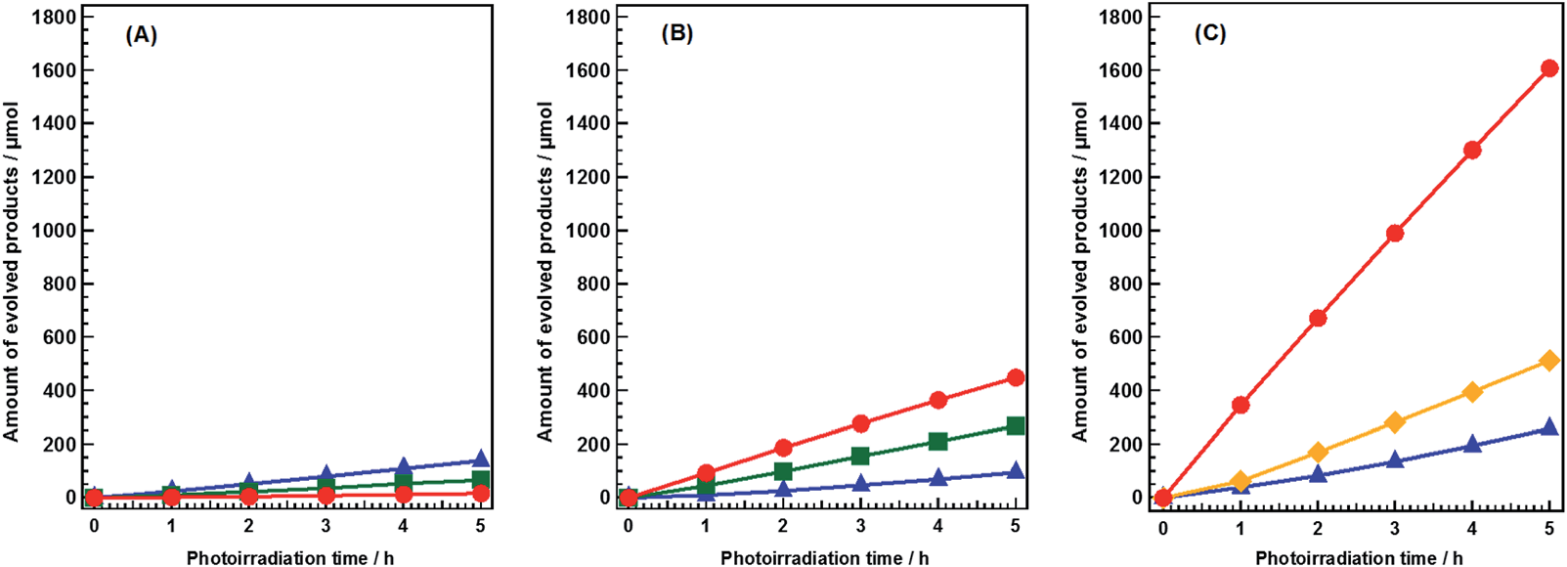
Time courses of CO (circle), O_2_ (square), N_2_ (lozenge), and H_2_ (triangle) evolutions during the photocatalytic conversion of CO_2_ over Ag-modified Sr_1.6_K_0.35_Na_1.45_Ta_5_O_15_. Amount of catalyst: 0.5 g; cocatalyst loading: 1.0 wt% Ag; light source: 400 W high-pressure Hg lamp; water volume: 1.0 L; CO_2_ flow rate: 30 mL min^–1^; additive: (A) none, (B) 0.1 M NaHCO_3_, or (C) 0.1 M NH_4_HCO_3_.

Fig. 1 shows the time courses of the photocatalytic conversion of CO_2_ in H_2_O and aqueous solutions of NaHCO_3_ and NH_4_HCO_3_. In pure H_2_O, only 16.8 μmol of CO was evolved after 5 h of photoirradiation (Fig. 1A), and the main reduction product was H_2_ (139.0 μmol). These results were consistent with previous reports.^25,29^ In this system, overall water splitting proceeded more readily than CO_2_ reduction, resulting in the generation of H_2_ as the major product, rather than CO. The amount of CO evolved in 0.1 M aqueous NaHCO_3_ solution after 5 h of photoirradiation (448.7 μmol) was 26.7 times higher than that evolved in pure H_2_O (Fig. 1B). However, the formation of H_2_ (94.7 μmol) was not significantly affected by NaHCO_3_. Thus, NaHCO_3_ greatly enhanced the conversion of CO_2_ to CO without affecting the water splitting process.^25^ In both pure H_2_O and 0.1 M aqueous NaHCO_3_, stoichiometric amounts of O_2_ were evolved continuously during the reaction, implying that H_2_O functioned as an electron donor in the reduction of CO_2_. Moreover, the evolution of CO increased dramatically in 0.1 M aqueous NH_4_HCO_3_ solution; 1600 μmol (1.6 mmol) of CO was evolved after 5 h of photoirradiation (Fig. 1C). This is 94.2 times greater than the amount evolved in pure H_2_O. The selectivity of the reaction toward CO evolution was calculated and the details were shown in ESI.† The selectivity toward CO evolution in 0.1 M aqueous NH_4_HCO_3_ (86.2%) was similar to that in aqueous NaHCO_3_ (82.5%). The production of gaseous products was negligible in blank tests conducted without either a catalyst or photoirradiation (Fig. S2A and B†). Thus, both are necessary for the photocatalytic conversion of CO_2_ to CO to proceed. Without Ag cocatalyst, H_2_ was formed as main product (Fig. S2C†), both of N_2_ and O_2_ were detected as oxidation products, however, the amount of these gases was far beyond the stoichiometric amount. Tiny amount of CO was formed after 5 hour photoirradiation (14.9 μmol). Ag cocatalysts were important in photocatalytic conversation of CO_2_ to CO, which is thought to be the active sites. H_2_, CO, and N_2_ were obtained without a continuous CO_2_ flow (Fig. S2D†). However, H_2_ was generated as a major product, suggesting a very low selectivity toward CO evolution (less than 30%). This suggested that CO_2_ presence significantly increases the selectivity of the photocatalytic conversion of CO_2_ toward CO evolution in NH_4_HCO_3_ solution. NH_4_HCO_3_ can be formed directly by the absorption of CO_2_ in an aqueous solution of NH_3_; wherein H_2_ and CO can be produced from CO_2_ and NH_3_ *via* artificial photosynthesis. Thus, our designed system can achieve carbon capture and utilization (CCU) in a single process.

**Fig. 2:**
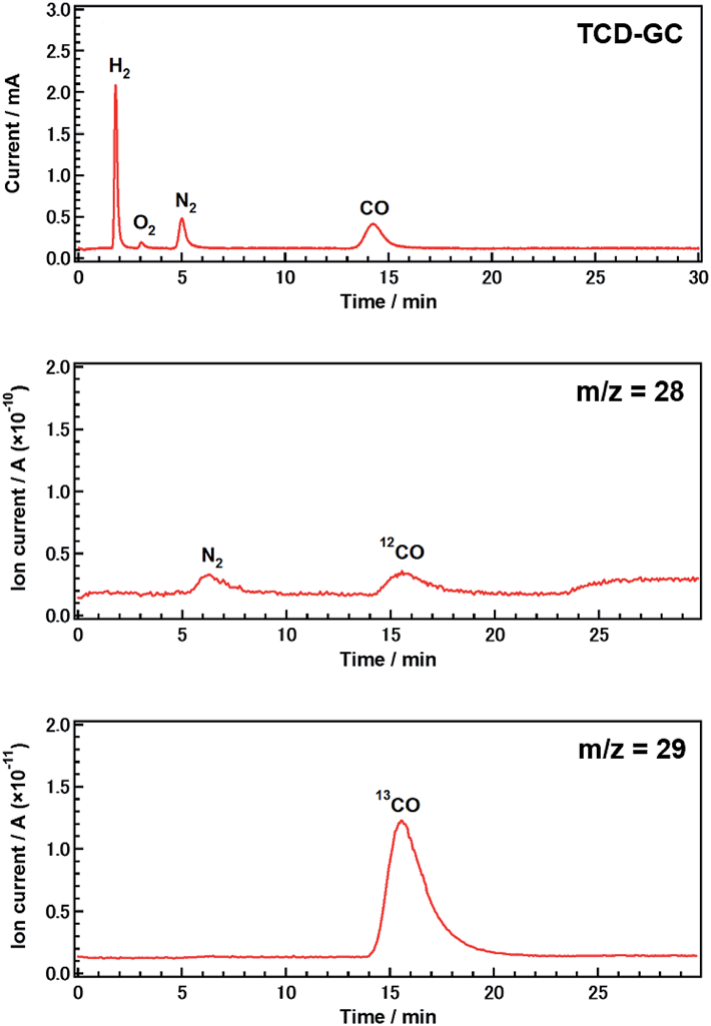
Gas chromatogram and mass spectra (*m*/*z* = 28 and 29) obtained during the photocatalytic conversion of ^13^CO_2_ using Ag-modified Sr_1.6_K_0.35_Na_1.45_Ta_5_O_15_. Amount of catalyst: 0.5 g; cocatalyst loading: 5.0 wt% Ag; light source: 400 W high-pressure Hg lamp; water volume: 1.0 L; CO_2_ flow rate: 30 mL min^–1^; additive: 0.5 M NH_4_HCO_3_.

N_2_, rather than O_2_, was generated as the oxidation product during photoirradiation in the presence of NH_4_HCO_3_ (Fig. 1C). This demonstrated that H_2_O did not function as an electron donor in this system. Instead, NH_3_ and/or NH_4_^+^ functioned as electron donors, because the Δ*G*° of NH_3_(aq) oxidation (18 kJ mol^–1^) is significantly lower than that of water oxidation (237 kJ mol^–1^). Analysis of the liquid phase showed that neither NO_2_^–^ nor NO_3_^–^ were present during photoirradiation (Fig. S3†). Other gaseous NO_*x*_ products, such as N_2_O and NO, were not detected by gas chromatography (GC). These results indicated that NH_3_ and/or NH_4_^+^ were oxidized only to N_2_ in this photocatalytic system. Hence, by using NH_4_HCO_3_, we succeeded in controlling the oxidation product, in addition to enhancing the conversion of CO_2_.

The ratio of electrons to holes consumed in the photocatalytic conversion of CO_2_ was calculated to be 2.0 after 1 h of photoirradiation (Fig. S4†). Given that the total number of electrons generated must be the same as the number of holes, this ratio indicates that significantly more electrons were consumed than holes in the initial stages of photoirradiation. We noted that the state of Ag was changed from metallic to Ag^+^ on the surface of catalyst measured by XPS (Fig. S5†), however, it might be not the main reason for the excess of electron consumption. We calculated that if all Ag^0^ was changed to Ag^+^ in the first hour, the consumed holes were still only 469 μmol, which was much less than the consumed electrons (770 μmol). NH_4_^+^ can be reduced to NH_3_ and ˙H by photogenerated electrons. Hydrazine (N_2_H_4_) has been determined to be an intermediate species in the photocatalytic decomposition of NH_3_ and/or NH_4_^+^ to H_2_ and N_2_ using Pt/TiO_2_.^14^ Stoichiometric amounts of products, including H_2_ and N_2_, were not obtained in the initial stages of photoirradiation due to the formation of hydrazine. In our system, it is also possible to form hydrazine at the beginning, however, hydrazine is reported to be reacted with CO_2_ to form zwitterionic intermediate and carbamate-type species,^30^ which made the detection of intermediate oxidation species much more difficult. Nevertheless, stoichiometric amounts of products were obtained after 2 h of photoirradiation (Fig. S4†), indicating that the total decomposition of NH_3_ and/or NH_4_^+^ occurred sooner.

The above results demonstrate that the highly efficient photocatalytic conversion of CO_2_ to CO was achieved in our system. The stoichiometric amounts of H_2_, N_2_ and CO generated indicated that NH_3_ and/or NH_4_^+^ functioned as electron donors in the photocatalytic conversion of CO_2_. Significantly greater photocatalytic activity was obtained using NH_3_ and/or NH_4_^+^, compared to reactions using H_2_O as an electron donor under the same conditions. NH_3_ and/or NH_4_^+^ are suitable for use in practical applications because NH_3_ is industrially produced in large quantities. Furthermore, in our photocatalytic system, NH_3_ and/or NH_4_^+^ can be completely decomposed to N_2_, which is an inert and non-toxic gas.

Table 1 shows the effects of NH_4_HCO_3_ concentration on the photocatalytic conversion of CO_2_. In pure H_2_O, overall water splitting proceeded as the dominant reaction. Hence, the evolution of CO was negligible (entry 1). When the photocatalytic reaction was carried out in 0.01 M aqueous NH_4_HCO_3_ the production of H_2_ resulting from water splitting was dramatically suppressed (entry 2). Because the oxidation of NH_3_ and/or NH_4_^+^ to N_2_ proceeds more readily than the oxidation of H_2_O to O_2_, the formation rate of O_2_ in 0.01 M aqueous NH_4_HCO_3_ was less than half that in pure H_2_O. Even low concentrations of NH_4_HCO_3_ (0.01 M) significantly increased the formation rate of CO, indicating that the presence of NH_4_HCO_3_ is vital to achieving high photocatalytic activity. NH_4_HCO_3_ can also be used to increase the pH of the reaction solution, to offset the decrease in pH caused by the dissolution of CO_2_. With CO_2_ flowing, the pH of the reaction solution based on pure H_2_O was 3.95, which increased to 5.88 with the addition of 0.01 M NH_4_HCO_3_. Increasing the pH also increases the amount of CO_2_ that can be dissolved in the reaction solution.^31^ Generally, the formation rate of CO increases with increasing pH, because the reaction rate largely depends on the concentration of substrate. Therefore, the addition of NH_4_HCO_3_ contributed to the efficient conversion of CO_2_ and the good selectivity toward CO evolution. Increasing the concentration of NH_4_HCO_3_ from 0.01 M to 0.05 M completely suppressed the overall water splitting reaction, since only N_2_ was generated as an oxidation product (entry 3). The formation rate of CO increased with the concentration of NH_4_HCO_3_. Increasing the NH_4_HCO_3_ concentration from 0.1 M to 1.0 M increased the formation rate of CO to 550.7 μmol h^–1^ except the selectivity toward CO evolution decreased slightly, from 86.1% to 65.5% (entry 4 to 7). As previously discussed, NH_4_HCO_3_ can be synthesized by flowing CO_2_ through an aqueous solution of NH_3_. To determine whether NH_3_ functions as an electron donor under a flow of CO_2_, we carried out the photocatalytic conversion of CO_2_ in an aqueous solution of NH_3_ (entry 8). The formation rate of CO was 547.2 μmol h^–1^ in *ca.* 0.5 M aqueous NH_3_, indicating that NH_3_ functions efficiently as an electron donor under these conditions. The ratio of photogenerated electrons to holes (e^–^/h^+^) was estimated to be around 1.0 in reactions with high concentrations of aqueous NH_4_HCO_3_ after 5 h of photoirradiation. This further supports the hypothesis that NH_3_ and/or NH_4_^+^ function as effective electron donors during the photocatalytic conversion of CO_2_.

**Table 1:** Photocatalytic conversion of CO_2_ over Ag-modified Sr_1.6_K_0.35_Na_1.45_Ta_5_O_15_ with different additive concentrations. Amount of catalyst: 0.5 g; cocatalyst loading: 1.0 wt% Ag; light source: 400 W high-pressure Hg lamp; water volume: 1.0 L; CO_2_ flow rate: 30 mL min^–1^

Entry	NH_4_HCO_3_ ^*a*^/M	Formation rate^*b*^/μmol h^–1^				Selec.^*c*^ (%)	e^–^/h^+^ ^*d*^
		H_2_	O_2_	N_2_	CO		
1	0	35.9	16.3	Trace	3.6	9.2	1.21
2	0.01	16.9	7.0	12.3	54.5	76.3	1.17
3	0.05	23.8	Trace	42.3	146.7	86.1	1.34
4	0.1	48.4	Trace	94.3	270.4	84.8	1.13
5	0.5	119.8	Trace	193.6	512.9	81.1	1.09
6	0.8	175.4	Trace	213.3	520.0	74.8	1.09
7	1.0	290.1	Trace	258.1	550.7	65.5	1.09
8^*e*^	—	235.0	Trace	244.9	547.2	70.0	1.06

^*a*^Additive concentration used for CO_2_ conversion.

^*b*^Formation rate after 5 h of irradiation.

^*c*^Selectivity toward CO evolution.

^*d*^Ratio of consumed electrons to holes after 5 h of irradiation.

^*e*^0.5 M aqueous NH_3_ solution was used as the additive, instead of NH_4_HCO_3_.

To confirm that CO evolution originated from CO_2_ introduced in the gas phase, rather than from carbon contaminants, we conducted an isotopic labeling experiment. Fig. 2 shows mass spectra (*m*/*z* = 28 and 29) obtained during the photocatalytic conversion of ^13^CO_2_ in 0.5 M aqueous NH_4_HCO_3_ over Ag-modified Sr_1.6_K_0.35_Na_1.45_Ta_5_O_15_ after 0.5 h of photoirradiation. Gaseous samples were introduced into a mass spectrometer (MS) after separation by thermal conductivity detector-gas chromatography (TCD-GC). CO was observed in both the gas chromatogram and the mass spectra. The peak positions in the mass spectra were consistent with those in the chromatogram. The major product was ^13^CO, rather than ^12^CO. The presence of a small amount of ^12^CO may be due to the direct decomposition of NH_4_HCO_3_ since the decomposition of NH_4_HCO_3_ was observed in samples without a CO_2_ flow (Fig. S2D†). The amount of ^13^CO estimated by mass spectrometry was approximately equal to the amount of CO determined using a flame ionization detector (FID-GC) (Fig. S6†). These results demonstrate that CO was predominantly generated from CO_2_ introduced in the gas phase, rather than from other carbon resources.

The recycle test was also performed to confirm the stability and durability of our catalyst and system using the Sr_1.6_K_0.35_Na_1.45_Ta_5_O_15_ photocatalyst repeatedly for three times under the same conditions (Fig. S7†). In the second cycle, there is a slight loss by *ca.* 10% of CO evolution activity during 5 h photoirradiation as compared to the first run, however, the evolution of H_2_ showed no obvious changes. The slight loss of activity should be due to the change of Ag cocatalyst (Fig. S5†).^29^ The photocatalytic activity of CO, N_2_, and H_2_ were stabilized at *ca.* 0.5, 0.19 and 0.07 mmol h^–1^, respectively, during the second and third runs. The structure of catalyst itself was stable during the three cycles (Fig. S8†). These results suggested that the photocatalyst and the system exhibit favorable stability to form CO, N_2_, and H_2_ during the photocatalytic conversion of CO_2_.

To confirm the versatility of NH_4_HCO_3_ as a general electron donor in photocatalytic reactions, we carried out the photocatalytic conversion of CO_2_ in aqueous NH_4_HCO_3_ solution over 4 types of photocatalysts. All these photocatalysts have been already reported to show good activity and high selectivity toward CO evolution in the photocatalytic conversion of CO_2_ using H_2_O as an electron donor.^25,32–34^ As shown in Table 2, all the photocatalysts showed good activity for conversion of CO_2_ and high selectivity toward CO evolution. The activities of the photocatalysts were significantly increased in aqueous NH_4_HCO_3_ solution, compared with their reported activities in pure H_2_O or aqueous NaHCO_3_.^32–34^ N_2_ was detected as the only oxidation product and the e^–^/h^+^ ratio was approximately equal to 1.0. These results indicated that NH_3_ and/or NH_4_^+^ was easily decomposed to N_2_ gas by the photocatalysts tested.

**Table 2:** Photocatalytic conversion of CO_2_ over Ag-modified catalysts in aqueous NH_4_HCO_3_ solution. Amount of catalyst: 0.5 g; cocatalyst loading: 5.0 wt% Ag; light source: 400 W high-pressure Hg lamp; water volume: 0.95 L; CO_2_ flow rate: 30 mL min^–1^; additive: 0.5 M NH_4_HCO_3_

Entry	Catalyst	Formation rate^*a*^/μmol h^–1^			Selec.^*b*^ (%)	e^–^/h^+^ ^*c*^
		H_2_	N_2_	CO		
1	ZnGa_2_O_4_/Ga_2_O_3_	125.2	191.4	532.0	80.9	1.14
2	ZnGa_2_O_4_	39.4	94.2	305.4	88.6	1.22
3	La_2_Ti_2_O_7_	5.9	17.4	41.6	87.6	0.91
4	SrO/Ta_2_O_5_	2.71	11.5	42.9	94.1	1.32

^*a*^Formation rate after 5 h of irradiation. O_2_ was not detected in any of the samples.

^*b*^Selectivity toward CO evolution.

^*c*^Ratio of consumed electrons to holes after 5 h of irradiation.

## Conclusions

We designed a highly efficient process for the photocatalytic conversion of CO_2_ to CO in aqueous NH_4_HCO_3_ solution. The stoichiometric formation of CO, H_2_, and N_2_ indicated that NH_3_ and/or NH_4_^+^ were consumed as electron donors, instead of H_2_O. NH_4_HCO_3_ was determined to be an effective electron donor for the photocatalytic conversion of CO_2_, whereby CO_2_ can be captured, stored, and efficiently converted into CO. This novel inorganic additive is suitable for use in carbon capture and utilization process. This new process is a promising way to control the conversion of CO_2_ to CO and efficiently produce H_2_ and CO.
