# Supplementary material for: Efficient photocatalytic carbon monoxide production from ammonia and carbon dioxide by the aid of artificial photosynthesis
Source: Chem Sci. 2017 Jun 19;8(8):5797–801. doi: 10.1039/c7sc01851g (PMC5625286; doi:10.1039/c7sc01851g)
Supplement: Supplementary file 1 [file SC-008-C7SC01851G-s001.pdf]

Electronic Supplementary Material (ESI) for Chemical Science.
This journal is © The Royal Society of Chemistry 2017

Supporting information

Efficient photocatalytic carbon monoxide production from ammonia and carbon dioxide by the aid of artificial photosynthesis

Zeai Huang,[a] Kentaro Teramura,[a,b]* Hiroyuki Asakura,[a,b] Saburo Hosokawa,[a,b] Tsunehiro Tanaka[a,b]*

[a]Department of Molecular Engineering, Graduate School of Engineering, Kyoto University, Kyoto 615-8510, Japan

[b]Elements Strategy Initiative for Catalysts and Batteries, Kyoto University, Kyoto 615-8510, Japan

Corresponding Author

*Dr. Kentaro Teramura. Tel: +81-75-383-2559, Fax: +81-75-383-2561, E-mail address: teramura@moleng.kyoto-u.ac.jp;

*Professor Tsunehiro Tanaka. Tel: +81-75-383-2558, Fax: +81-75-383-2561, E-mail address: tanakat@moleng.kyoto-u.ac.jp

Experiment section

Catalysts preparation

$Sr_{1.6}K_{0.35}Na_{1.45}Ta_5O_{15}$ was fabricated using a flux method. KCl (99.8%, Wako) and NaCl (99.9%, Wako) in a molar ratio of 55:45 were used as flux. A stoichiometric mixture of K_2CO_3 (99.5%, Wako), $SrCO_3$ (99.9%, Wako), and Ta_2O_5 (99.9%, Kojundo) was ground with flux in an Al_2O_3 mortar for 5 min. The total mass ratio of flux to precursors (K_2CO_3, $SrCO_3$, and Ta_2O_5) was fixed at 1.0. The mixture was transferred to a 50 mL Al_2O_3 crucible and calcined at 1173 K for 3 h in air. After cooling to room temperature, the mixture was washed three times with distilled water at 358 K, to remove any residual salts, then dried in air at 353 K. Elemental analysis was conducted using ICP-OES (iCAP7400 Duo, Thermo Fisher Scientific, Inc.) to determine the chemical formula of the catalyst; $Sr_{1.6}K_{0.35}Na_{1.45}Ta_5O_{15}$. Other photocatalysts were fabricated according to the reported papers in our previous works.[1-4] The Ag cocatalyst was deposited onto the fabricated samples using a chemical reduction method. Specifically, an aqueous $NaPH_2O_2$ solution (0.33 M) was added to a 50-mL suspension containing the photocatalyst (1.5 g) and $AgNO_3$ (0.1 M). After stirring at 358 K for 1.5 h, the suspension was filtered and the catalyst was dried in air at room temperature before use.

Characterization

The structures and crystallinities of the samples were characterized by X-ray diffraction (XRD) using a Rigaku Multiflex powder X-ray diffractometer. SEM images were obtained using a field emission scanning electron microscope (FE-SEM, SU-8220, Hitachi High-Technologies). The XPS measurement was acquired using an X-ray photoelectron spectrometer (ESCA 3400, Shimadzu Corp.), and calibrated by the peak that can be assigned to C 1s.

Photocatalytic activity test

Photocatalytic conversions of CO_2 by H_2O were carried out in a flow system using an inner-irradiation-type reaction vessel at room temperature and ambient pressure. The photocatalyst (0.5 g) was dispersed in aqueous solution (1.0 L) with different additives, and CO_2 gas (99.999%) was bubbled into the solution at a flow rate of 30 mL min^{-1}. The suspension was irradiated using a 400 W high-pressure Hg lamp with a quartz filter connected to a water cooling system. The gaseous products generated in the reaction (such as H_2, O_2, N_2, and CO) were analyzed by thermal conductivity detector-gas chromatography (TCD-GC) using a GC-8A chromatograph (Shimadzu Corp.) equipped with a Molecular Sieve 5A column using Ar as the carrier gas, and by flame ionization detector-gas chromatography (FID-GC) with a methanizer and a ShinCarbon ST column using N_2 as the carrier gas. In the isotopic experiment, $^{12}CO_2$ gas was replaced with $^{13}CO_2$. The formation of ^{13}CO and ^{12}CO was analyzed using a quadrupole mass spectrometer (BEL Japan, Inc., BEL Mass) combined with TCD-GC using Ar as the carrier gas.

Calculation of selectivity toward CO evolution and consumed e^-/h^+

In a photocatalytic reaction, charge separation takes place inside a bulk of photocatalyst, in which electron and hole pairs are generated. Electrons and holes that are not deactivated by recombination move separately to the surface of the photocatalyst. As a result, reduction and oxidation occur at the surface electrons and holes, respectively.

- ✓ **Reduction process**

  Two reduction reactions take place in our system: (1) reduction of CO_2 into CO and (2) production of H_2 for the photocatalytic conversion of CO_2 by H_2O. These are both 2-electrons reduction processes, as shown below.

  - ➢ Reduction of CO_2: $CO_2 + 2H^+ + 2e^- \rightarrow CO + H_2O$ (1)
  - ➢ Production of H_2: $2H^+ + 2e^- \rightarrow H_2$ (2)

In the photocatalytic conversion of CO_2 by H_2O, H_2 can be produced from water splitting, which competes with the reduction of CO_2. However, this H_2 production is not a favorable reaction. Therefore, the fraction of electrons used for the reduction of CO_2 should be considered. The selectivity of the generated electrons toward CO evolution can be calculated using Eq. 3.

- Selectivity (%) = $100 \times 2\boldsymbol{R}_{CO} / (2\boldsymbol{R}_{CO} + 2\boldsymbol{R}_{H2})$ (3)

where $\boldsymbol{R}_{CO}$ and $\boldsymbol{R}_{H2}$ represent the formation rates of CO and H_2, respectively.

✓ **Oxidation process**

Generally, O_2 is generated when H_2O functions as an electron donor, as in Eq. 4.

- Oxidation of H_2O: $2H_2O \rightarrow O_2 + 4H^+ + 4e^-$ (4)

The oxidation of H_2O is a typical 4-electron oxidation process. We found that NH_3 and/or NH_4^+ functioned as electron donors in our system, as shown below.

- Oxidation of NH_3: $2NH_3 \rightarrow N_2 + 6H^+ + 6e^-$ (5)
- Oxidation of NH_4^+: $2NH_4^+ \rightarrow N_2 + 8H^+ + 6e^-$ (6)

Six electrons are produced in the oxidation of either NH_3 or NH_4^+ to N_2. Thus, six holes should be consumed. The formations of H_2 and CO are both 2-electron reduction processes, as shown in Eqs. 1 and 2. Thus, the ratio of electrons consumed by holes can be calculated from the formation rates of each gas, as shown below.

- Consumed $e^-/h^+ = (2\boldsymbol{R}_{CO} + 2\boldsymbol{R}_{H2}) / (4\boldsymbol{R}_{O2} + 6\boldsymbol{R}_{N2})$

where $\boldsymbol{R}_{CO}$, $\boldsymbol{R}_{H2}$, $\boldsymbol{R}_{O2}$, and $\boldsymbol{R}_{N2}$ represent the formation rates of CO, H_2, O_2, and N_2, respectively. The number of electrons generated by charge transfer is strictly equal to that of holes. When the photocatalytic reaction proceeds stoichiometrically, the ratio of electrons to holes will be equal to 1 ($e^-/h^+ = 1$).

✓ **Total process**

The total chemical formulae in cases where only NH_3 or NH_4^+ acts as the electron donor (therefore, O_2 is not evolved) are as follows:

- $xCO_2 + yNH_3 \rightarrow (y/2)N_2 + (1.5y - x)H_2 + xCO + xH_2O$ (7)
- $xCO_2 + yNH_4^+ \rightarrow (y/2)N_2 + (1.5y - x)H_2 + xCO + xH_2O + yH^+$ (8)

For example, if no H_2 is generated, 1 mol of N_2, 3 mol of CO, and 3 mol of H_2O will be generated from 2 mol of CO_2 and 3 mol of NH_3 or NH_4^+. The ratio of H_2 to CO (H_2/CO) can be controlled by tuning the decomposition of NH_3 or NH_4^+ in the photocatalytic system, for instance, by changing the concentration of NH_4HCO_3.

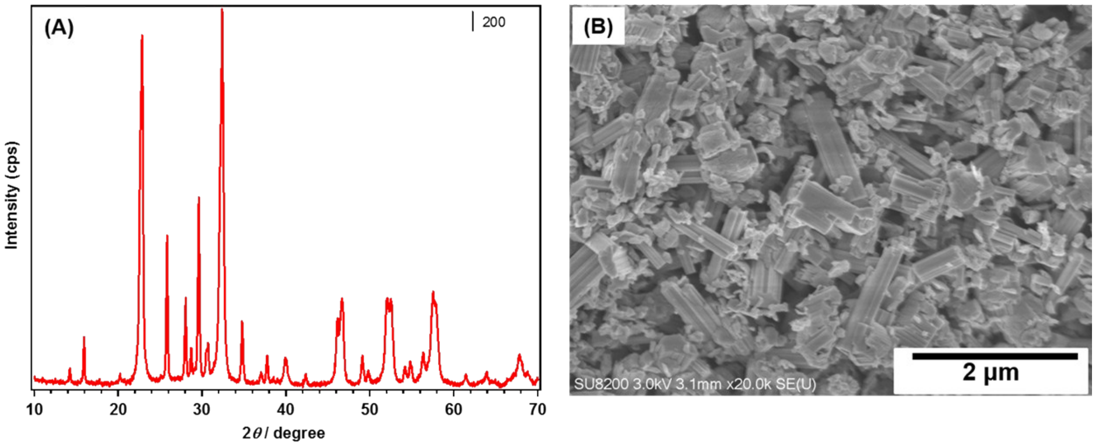

Figure S1 XRD pattern (A) and SEM image (B) of $Sr_{1.6}K_{0.35}Na_{1.45}Ta_5O_{15}$ prepared using a modified flux method and calcined at 1173 K for 3 h.

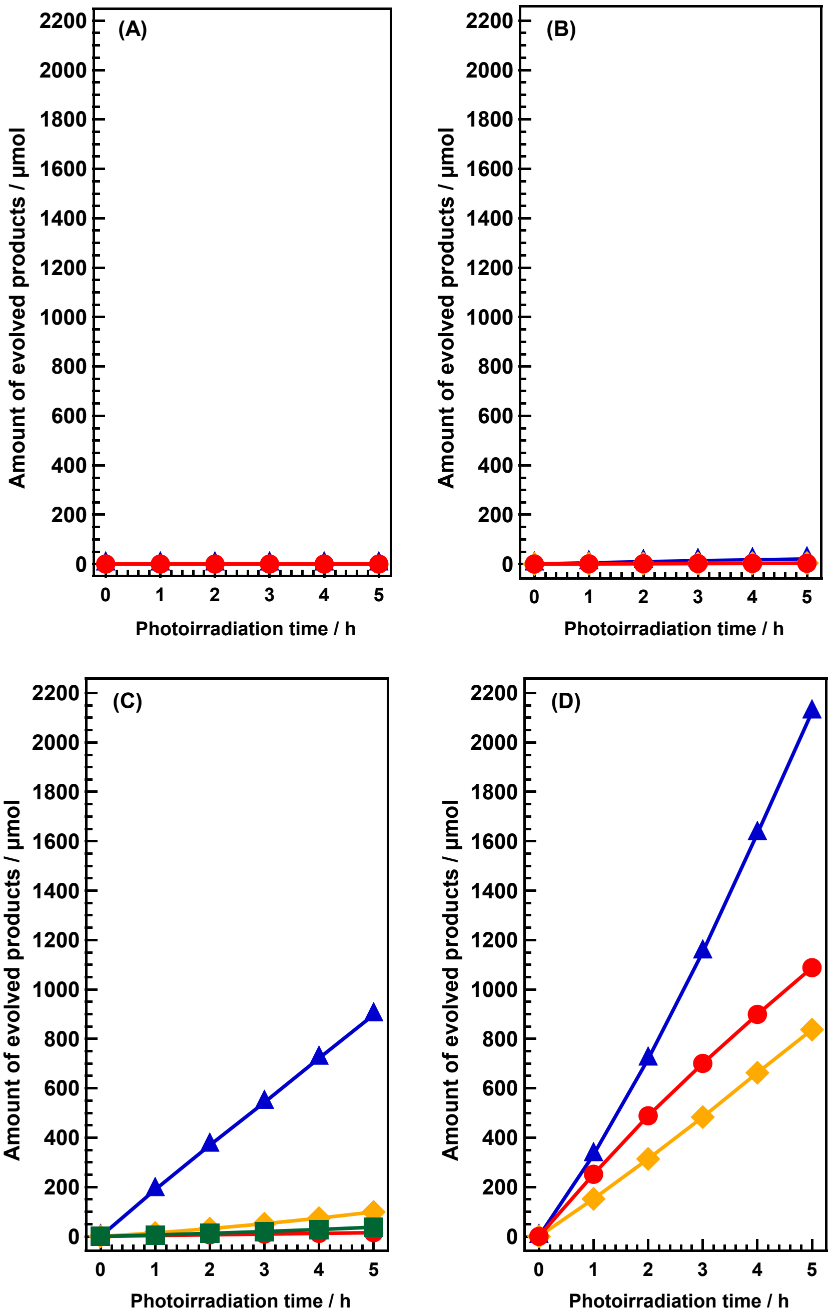

Figure S2 Time courses of CO (circle), O_2 (square), N_2 (lozenge), and H_2 (triangle) evolutions during the photocatalytic conversion of CO_2 over Ag-modified $Sr_{1.6}K_{0.35}Na_{1.45}Ta_5O_{15}$. (A) Without photocatalyst, (B) without photoirradiation, (C) without Ag cocatalyst, and (D) without CO_2 flow.

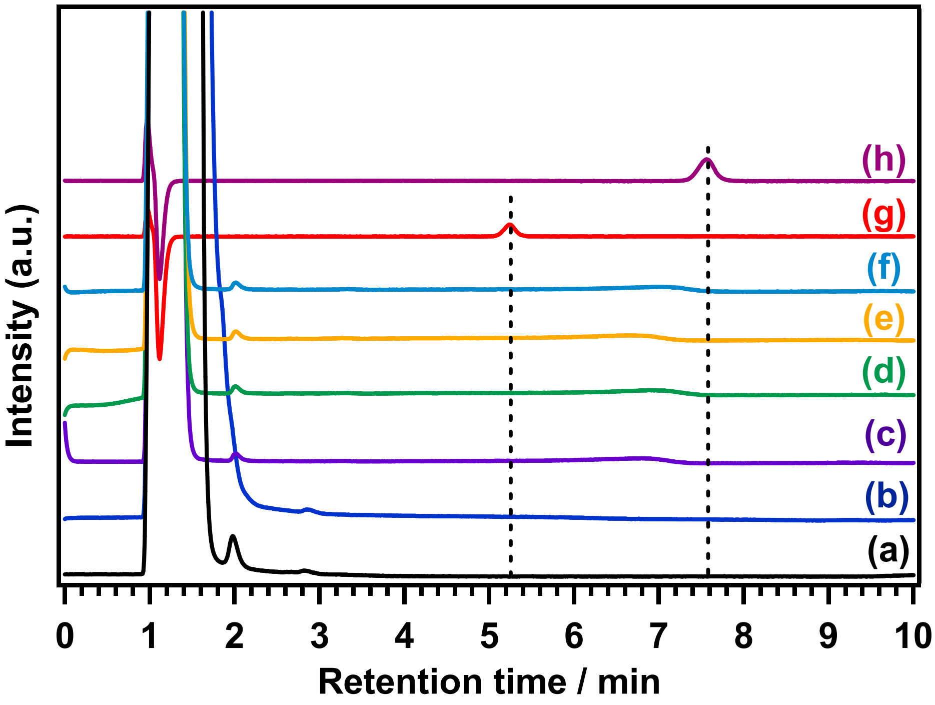

Figure S3 HPLC chromatograms of reaction solutions after photoirradiation for (a) 0.5 h, (b) 1.0 h, (c) 2.0 h, (d) 3.0 h, (e) 4.0 h, and (f) 5.0 h over 1.0 wt.% Ag-modified $Sr_{1.6}K_{0.35}Na_{1.45}Ta_5O_{15}$. (g) and (h) are 100 ppm $NaNO_3$ and $NaNO_2$, respectively.

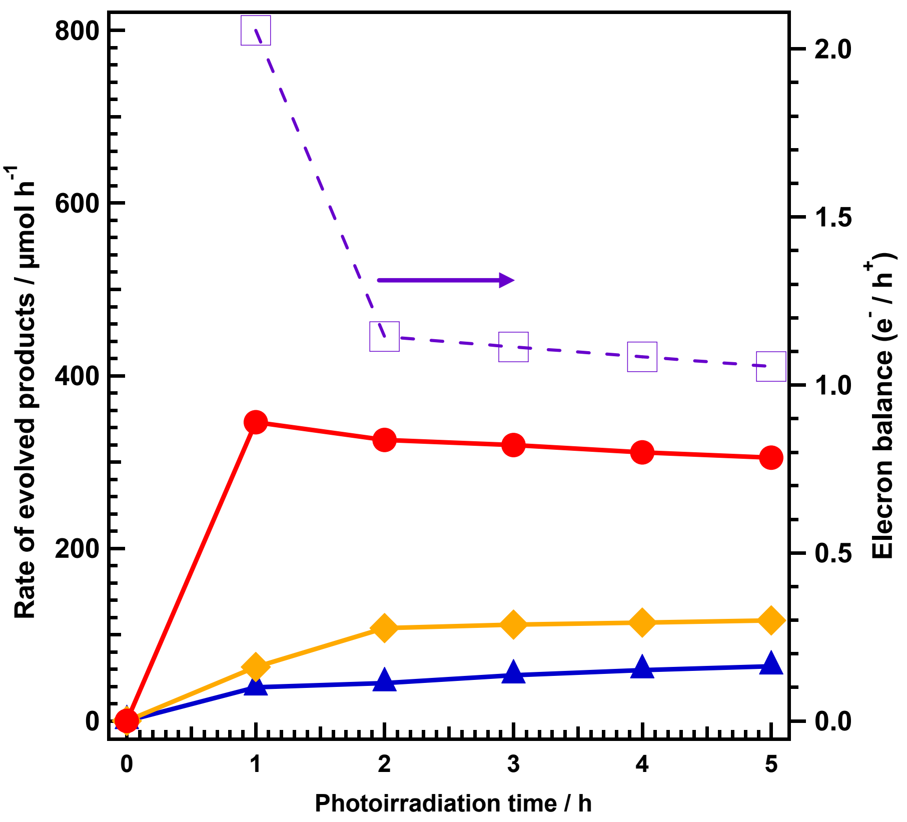

Figure S4 Time course of CO (circle), N_2 (lozenge), and H_2 (triangle) evolutions and electron balance (e^-/h^+) (square) during the photocatalytic conversion of CO_2 over 1.0 wt.% Ag-modified $Sr_{1.6}K_{0.35}Na_{1.45}Ta_5O_{15}$.

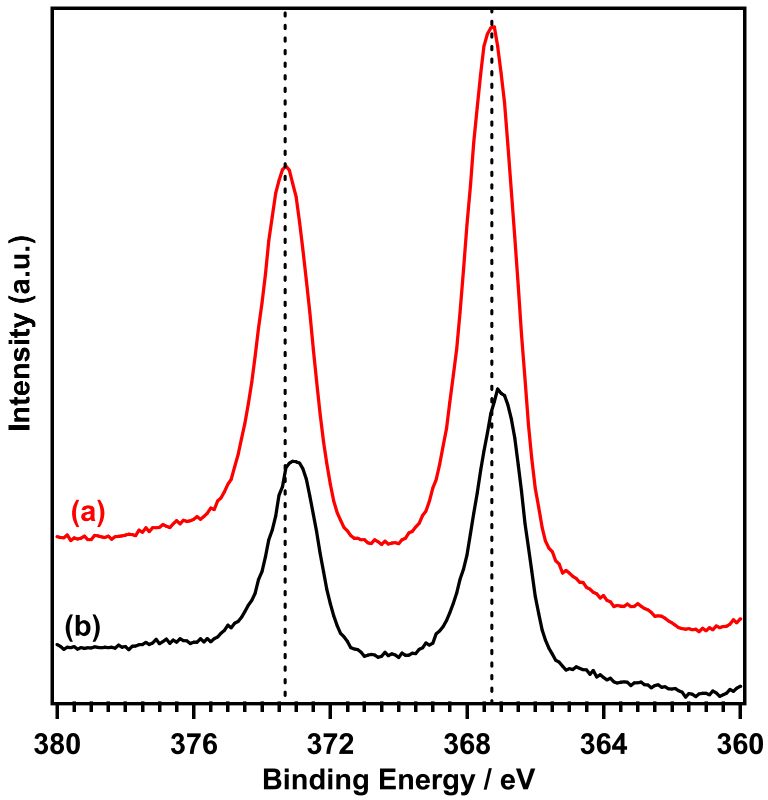

Figure S5 X-ray photoelectron spectra of Ag3d before (a) and after (b) the photocatalytic conversion of CO_2 over $Sr_{1.6}K_{0.35}Na_{1.45}Ta_5O_{15}$.

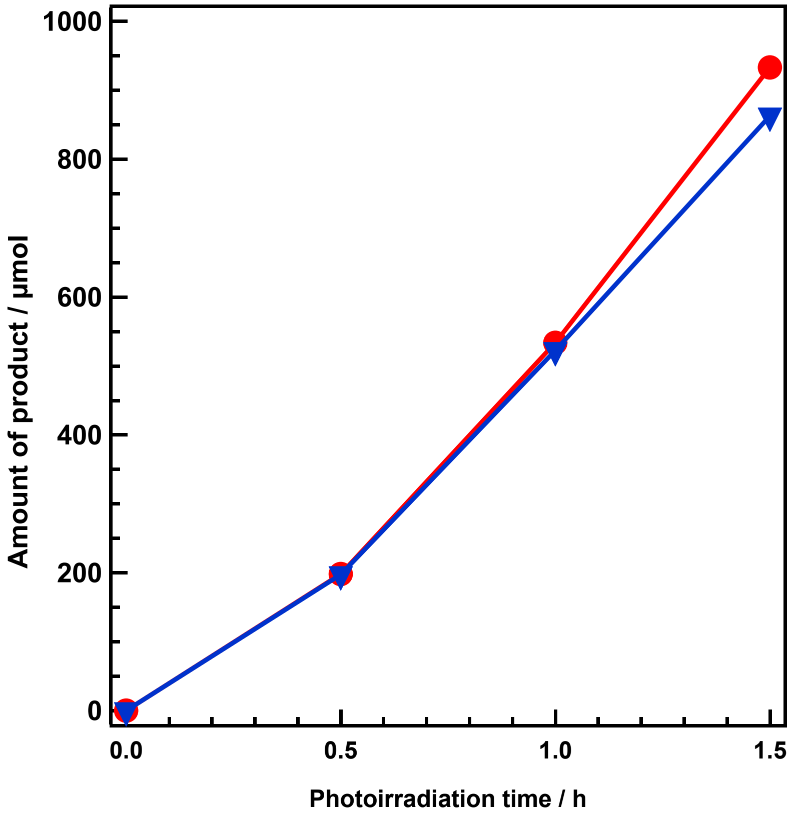

Figure S6 Time course of CO (circle) and ^{13}CO (triangle) determined by FID-GC and MS, respectively, during the photocatalytic conversion of CO_2 over 5.0 wt.% Ag-modified $Sr_{1.6}K_{0.35}Na_{1.45}Ta_5O_{15}$.

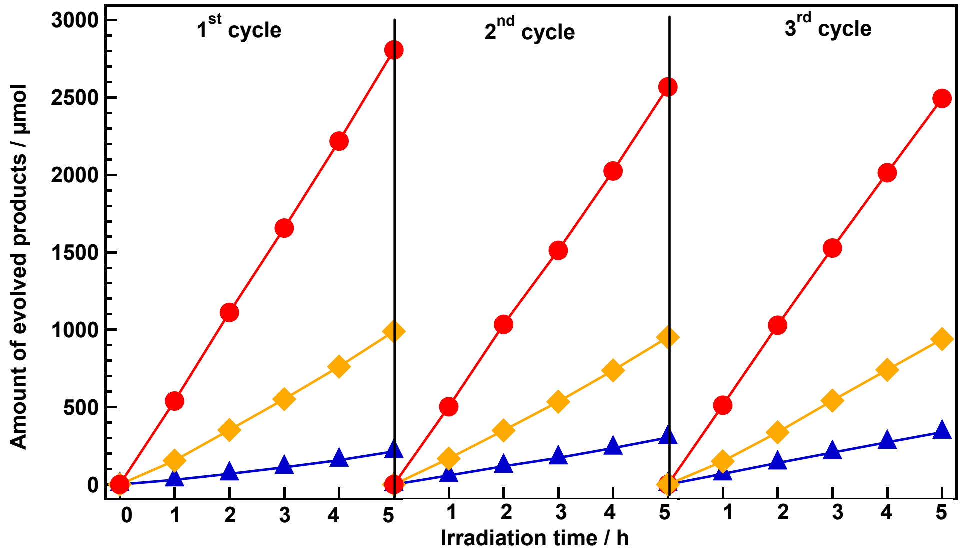

Figure S7 Time course of CO (circle), N_2 (lozenge), and H_2 (triangle) evolutions during three cycles of the photocatalytic conversion of CO_2 over Ag-modified $Sr_{1.6}K_{0.35}Na_{1.45}Ta_5O_{15}$. Amount of catalyst: 0.5 g; cocatalyst loading: 5.0 wt.% Ag; light source: 400 W high-pressure Hg lamp; water volume: 1.0 L; CO_2 flow rate: 30 mL min^{-1}; additive: 0.5 M NH_4HCO_3.

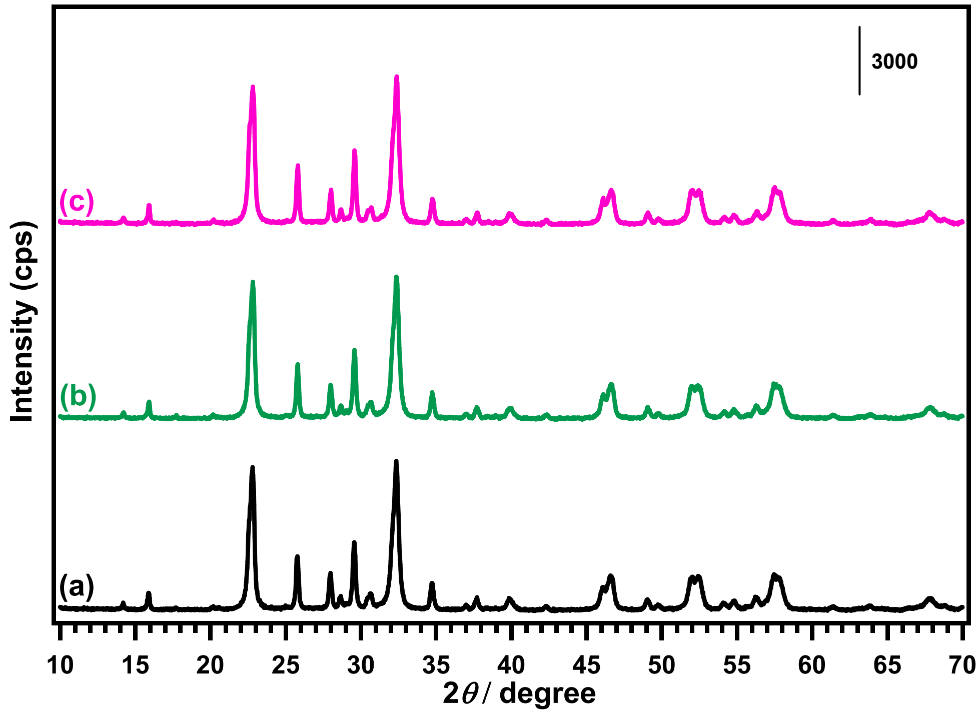

Figure S8 XRD patterns of $Sr_{1.6}K_{0.35}Na_{1.45}Ta_5O_{15}$ after (a) first, (b) second and (c) third cycle.

Refences

1. Wang, Z.; Teramura, K.; Hosokawa, S.; Tanaka, T., *Appl Catal B-Environ* **2015,** *163*, 241-247.
2. Teramura, K.; Tatsumi, H.; Wang, Z.; Hosokawa, S.; Tanaka, T., *Bulletin of the Chemical Society of Japan* **2015,** *88* (3), 431-437.
3. Teramura, K.; Wang, Z.; Hosokawa, S.; Sakata, Y.; Tanaka, T., *Chemistry–A European Journal* **2014,** *20* (32), 9906-9909.
4. Wang, Z.; Teramura, K.; Hosokawa, S.; Tanaka, T., *J. Mater. Chem. A* **2015,** *3* (21), 11313-11319.
